# Systematic Investigations on the Metabolic and Transcriptomic Regulation of Lactate in the Human Colon Epithelial Cells

**DOI:** 10.3390/ijms23116262

**Published:** 2022-06-02

**Authors:** Chongyang Huang, Huanzhou Xu, Xin Zhou, Maili Liu, Jing Li, Chaoyang Liu

**Affiliations:** 1 State Key Laboratory of Magnetic Resonance and Atomic and Molecular Physics, Wuhan Institute of Physics and Mathematics, Innovation Academy for Precision Measurement Science and Technology, Chinese Academy of Sciences, Wuhan 430071, China; chyhuang999@apm.ac.cn (C.H.); xinzhou@wipm.ac.cn (X.Z.); ml.liu@wipm.ac.cn (M.L.); 2 Department of Pediatrics, Division of Infectious Diseases, University of Florida College of Medicine, Gainesville, FL 32608, USA; huanzhouxu@163.com; 3 University of Chinese Academy of Sciences, Beijing 100049, China; 4 Optics Valley Laboratory, Wuhan 430074, China; 5 Wuhan Botanical Garden, Chinese Academy of Sciences, Wuhan 430074, China

**Keywords:** lactate, mitochondrial dysfunction, NMR-based metabolomics, transcriptomics, one-carbon metabolism, polyol pathway

## Abstract

Lactate, primarily produced by the gut microbiota, performs as a necessary “information transmission carrier” between the gut and the microbiota. To investigate the role of lactate in the gut epithelium cell–microbiota interactions as a metabolic signal, we performed a combinatory, global, and unbiased analysis of metabolomic and transcriptional profiling in human colon epithelial cells (Caco-2), using a lactate treatment at the physiological concentration (8 mM). The data demonstrated that most of the genes in oxidative phosphorylation were significantly downregulated in the Caco-2 cells due to lactate treatment. Consistently, the levels of fumarate, adenosine triphosphate (ATP), and creatine significantly decreased, and these are the metabolic markers of OXPHOS inhibition by mitochondria dysfunction. The one-carbon metabolism was affected and the polyol pathway was activated at the levels of gene expression and metabolic alternation. In addition, lactate significantly upregulated the expressions of genes related to self-protection against apoptosis. In conclusion, lactate participates in gut–gut microbiota communications by remodeling the metabolomic and transcriptional signatures, especially for the regulation of mitochondrial function. This work contributes comprehensive information to disclose the molecular mechanisms of lactate-mediated functions in human colon epithelial cells that can help us understand how the microbiota communicates with the intestines through the signaling molecule, lactate.

## 1. Introduction

Lactate had been designated as a waste product of anaerobic glycolysis for nearly a century [1,2]. However, recent publications have demonstrated that lactate is an active molecule and plays a crucial role in multiple cellular processes including respiration regulation [3], immune response [4,5,6], neuronal memory allocation [7], and cancer growth [8,9]. In addition, lactate is a standard natural product of gut bacteria that is primarily fermented by the microbiome, such as lactic acid bacteria of the genera *Lactobacillus* and *Bifidobacterium* [10,11]. Lactate induces several modulations and profound impacts on various biological processes in the gut function [12,13] and diseases [14,15]. Previous studies have reported that lactate has bioactive capacities that act in at least three independent ways [16], including (1) modulating gene expression by the modification of histone deacetylase (HDAC) activity [17], (2) triggering different signaling pathways by GPR81 [18,19], and (3) inducing changes in metabolic pathways [20]. In addition, lactate-utilizing bacteria play a crucial role in modulating the colonic metabolome [21].

Lactate is also a metabolic product of the host cells. High levels of lactate are used similarly in the gut, being converted into acetate, propionate, or butyrate [22]. The concentration of lactate in the gut is still highly controversial, primarily due to different detection methods, the complicated gut microbiota environment, and the high turnover rate of lactate in the intestine [23]. The balance between production by bacteria and by host tissues, microbial utilization, and host absorption makes up the final fecal concentration of lactate. Though lactate can be found in fermented foods at levels from about 5 to 100 mM [24,25], less than 3 mM of lactate can be detected [26]. Bacteria utilize a large amount of the lactate via metabolite cross-feeding in a healthy colon, and this induces a low lactate level in fecal matter [21]. However, a high lactate level often occurs in the gut. The maximum level of 8.5 (5.0 to 14.9) mM of gut luminal lactate was measured using microanalysis in an ischemia model [27]. Significantly higher levels of lactic acid (mean 8·4 mM) have been shown in breastfed babies when compared with formula-fed babies (mean 1·7 mM) at the age of one month [28]. Lactate concentrations up to approximately 100 mM have been associated with ulcerative colitis [29,30]. Previous evidence suggested that lactate may alter the pro-inflammatory response at doses ranging from 10 to 20 mM (10 mM of lactate significantly decreased the amount of TNF mRNA after 4 h at the transcriptional level, and 20 mM of lactate was needed to suppress IL-6 at a pro-inflammatory level [31]). From the receptor aspect, the selectivity of GPR81 on lactate is within a wide range of 0.1–30.0 mM [32,33]. Collectively, different concentrations of lactate result in different biological effects in the gut. Therefore, it is important to investigate the function of lactate at the physiological concentration.

The integration of transcriptome and metabolome will yield more reasonable insights in revealing the molecular pathways leading to the phenotype under lactate exposure in human colon epithelial cells that are primarily used as a model of the intestinal epithelial barrier [34]. The role of lactate in the live system has been discussed widely. However, an understanding of the molecular mechanism is still rare in Caco-2 cells from the metabolic and transcription aspects, and the tools and critical players are only beginning to be elucidated. Transcriptomics is used to identify genes that are differently expressed in many disease models and to investigate the mechanism at a molecular level [35]. Information regarding transcriptome and metabolome has significantly contributed to many research areas such as cancer [36] and obesity [37]. Additionally, metabolomics offers a holistic approach to understanding the phenotype of an organism based on those metabolites that represent molecular endpoints of gene expression and cell activity [38]. In this study, to provide comprehensive information at the molecular level to understand how lactate affects the intestine, we employ a combination of an unbiased and global metabolic and a transcriptional approach to investigate the effects of lactate treatment on the function of Caco-2 cells at the physiological concentration (8 mM).

## 2. Results

### 2.1. The Effect of Lactate on Caco-2 Cells Growth

**Figure 1:**
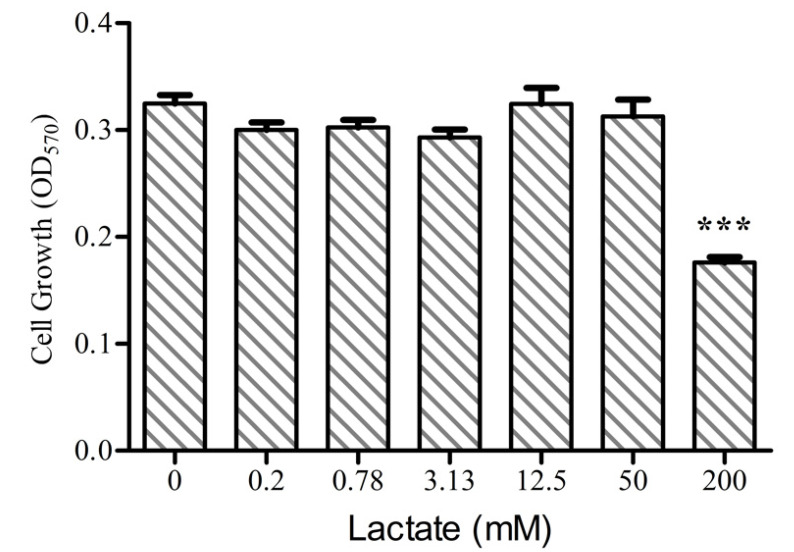
Determination of the cytotoxic activity of lactate on Caco-2 Cells using the MTT assay. N = 4, values are means ± SEM, *** *p* < 0.0001.

We selected the colon epithelial cells, since the colon harbors the highest diversity and density of microbiota in the gastrointestinal tract [39,40], including significant lactate producers, Bifidobacterium spp., and lactic acid bacteria such as Lactobacillus and Enterococcus spp [41]. To determine the nontoxic concentration of lactate on Caco-2 growth, a growth curve was made using the MTT assays. Compared with the control group, less than 50 mM of lactate had no impact on the Caco-2 cell growth, but 200 mM of lactate significantly inhibited the viability after 24 h of treatment (Figure 1, *p* < 0.0001). Another study reported that concentrations of lactate up to 20 mM did not affect cell growth [42].The physiological concentration in stool samples from healthy adults is approximately 8.5 mM [27,28], but concentrations up to 100 mM have been reported in gut disorders such as ulcerative colitis [43]. Therefore, we next investigated the effect of 8 mM of lactate treatment on the metabolomics and transcriptomics profiles of the Caco-2 cells.

### 2.2. Metabolite Assignments for the ^1^H NMR Spectroscopy

**Figure 2:**
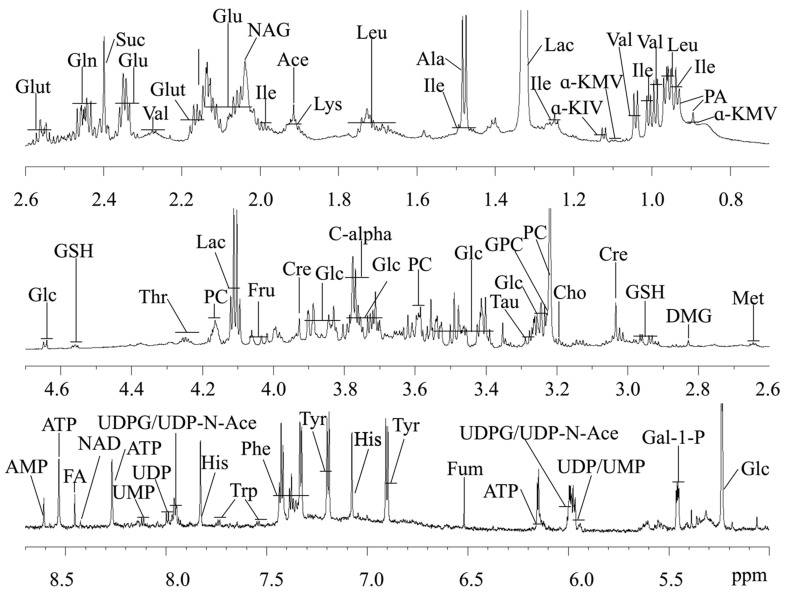
Typical ^1^H NMR spectra from the cell extracts. Keys: PA, Pantothenate; Ile, Isoleucine; Leu, Leucine; Val, Valine; α-KIV, α-Keto-isovalerate; α-KMV, α-keto-beta-methyl-valerate; Lac, Lactate; Ala, Alanine; Thr, Threonine; Glu, Glutamate; Gln, Glutamine; NAG, N-Acetyl-Glutamine; Suc, Succinate; Met, Methionine; DMG, Dimethylglycine; Cre, Creatine; Cho, Choline; PC, Phosphocholine; GPC, Glycerophosphocholine; Tau, Taurine; GSH, Glutathione; Glc, Glucose; Fru, Fructose; Gal-1-*p*, Galactose-1-phosphate; Tyr, Tyrosine; Phe, Phenylalanine; Fum, Fumaric acid; NAD^+^, Nicotinamide adenine dinucleotide; FA, Formate; His, Histidine; AMP, Adenosine monophosphate; ATP, Adenosine triphosphate; UMP, Uridine 5′-monophosphate; UDP, Uridine-5′-diphosphate; UDPG, UDP Glucuronate; UDP-*N*-Ace, UDP-*N*-acetylglucosamine; C-alpha, The central carbon atom in amino acids.

The typical ^1^H NMR spectra of the cell extracts obtained from the control group are shown in Figure 2. Metabolite assignments were conducted as described in the literature [44] and confirmed using the two-dimensional NMR spectra. The spectra of the cell extraction were dominated by numerous metabolites, as shown by the metabolite key in Figure 2. The signals included amino acids (e.g., glutamine, valine, isoleucine, and leucine), sugars (e.g., glucose and fructose), a range of organic acids (e.g., lactate, fumaric acid, and succinate), and nucleotides (e.g., adenosine monophosphate, adenosine triphosphate, uridine 5′-monophosphate, and uridine-5′-diphosphate) in the low field between 5.00 ppm and 9.4 ppm. The NMR resonance assignments and proton chemical shifts and their diversity are listed for the identification of chemical structures of these metabolites in Table 1.

### 2.3. Significantly Changed Metabolites Detected by High-Resolution ^1^H NMR

**Figure 3:**
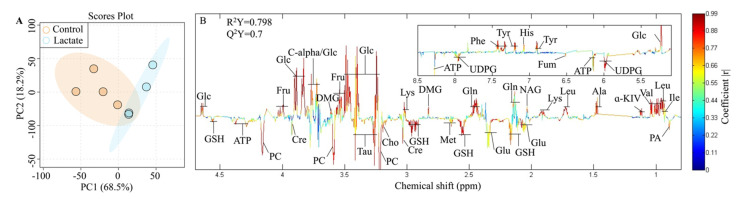
Differential metabolomics of the Caco-2 cells between the lactate and the control group. (**A**). Principal component analysis (PCA) scores of the complete metabolic profiles of all samples (N = 4). The light blue region is for the 95% confidence interval of the lactate treatment; the light orange portion is for the 95% confidence interval of the control group; and the light blue and orange dots are representative of the samples of the lactate and the control treatments, respectively. (**B**). Color-coded correlation coefficient loadings plots generated by comparing the spectra of the intracellular metabolites between the lactate and the control treatments.

Lactate plays a crucial role in rewiring the metabolic program in the gut. In our study, the PCA map showed two distinct clusters of the metabolome between lactate and the control treatment group, including 11 significantly decreased metabolites and 14 significantly increased metabolites, and there were no outliers outside of the 95% confidence interval (Figure 3A). Of these metabolites, the levels of fumarate, ATP, and creatine, which are the metabolic markers of mitochondria dysfunction, significantly reduced in Caco-2 cells with lactate treatment, compared with the control treatment (Figure 3B). Notably, lactate treatment induced an elevation in glucose, fructose and a reduction in glutathione (GSH) (Figure 3B), indicating an increase in glucose uptake and the activation of the polyol pathway. In addition, lactate treatment increased the levels of amino acids including leucine, lysine, phenylalanine, tyrosine, isoleucine, valine, and histidine in the cytosol (Figure 3B). Interestingly, the levels of methionine, taurine, choline, and phosphocholine were significantly reduced by the lactate treatment, while the level of dimethylglycine significantly increased, which are all linked with the one-carbon metabolism in the cell.

### 2.4. Lactate Modulated Transcriptome Signatures in Human Colon Epithelial Cells

**Figure 4:**
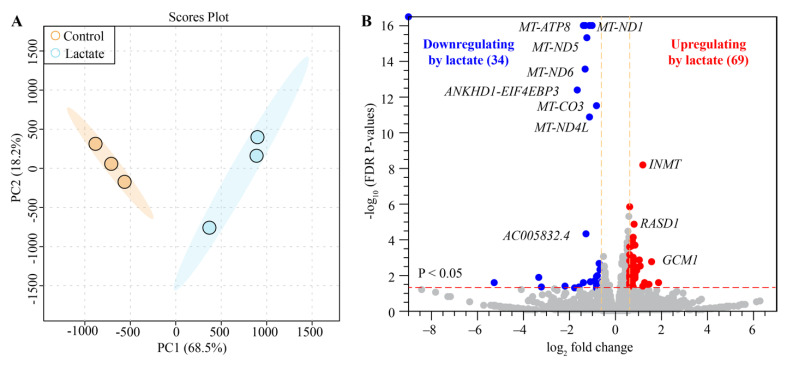
Comparison of the transcriptomes of the Caco-2 cells of the lactate and the control treatments. (**A**). Principal component analysis (PCA) scores of the complete expression profiles of all the samples (N = 3). The light blue region is for the 95% confidence interval of the lactate treatment; the light orange region is for the 95% confidence interval of the control group; and the light blue and orange dots are representative of the samples of the lactate and the control treatments, respectively. (**B**). Volcano plot showing the differentially expressed genes (DEGs) in the Caco-2 Cells of the lactate and the control treatments. The FDR-corrected *p* < 0.05; the absolute fold change >= 1.5. The blue dots are the downregulated DEGs, and the red dots are the upregulated DEGs caused by the lactate treatment. The gray dots are the genes with no difference in expression.

We performed high-throughput RNA sequencing to determine the effects of lactate on the transcriptional program of Caco-2 cells. The PCA map showed two distinct clusters of the global transcriptome between the lactate and control treatments, and there were no outliers outside of the 95% confidence interval (Figure 4A). We identified 103 top genes that were significantly changed between the two groups. Of which, 34 were downregulated and 69 upregulated (FDR-corrected *p* < 0.05, absolute fold change ≥1.5) by the lactate treatment compared with the control treatment (Figure 4B).

**Figure 5:**
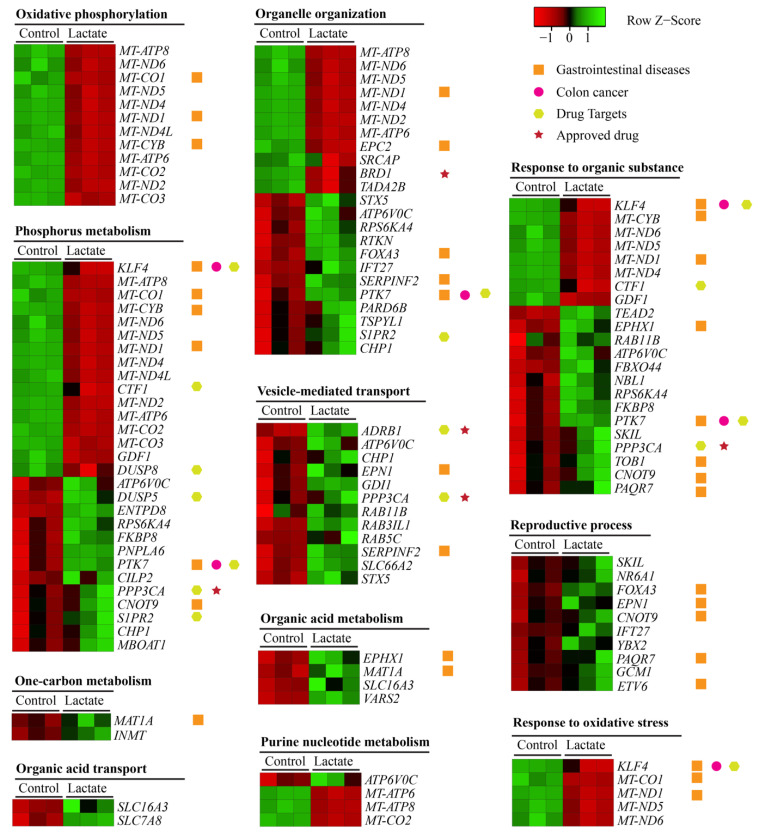
Supervised heat maps of genes in the enriched gene sets. The gene set enrichment analyses determined that the enriched gene sets and were performed using supervised hierarchical clustering in Heatmapper including DEGs with an FDR-corrected *p* < 0.05 and fold change ≥1.5, upregulated (red) gene, and downregulated (green) gene in the lactate vs. the control treatments.

**Figure 6:**
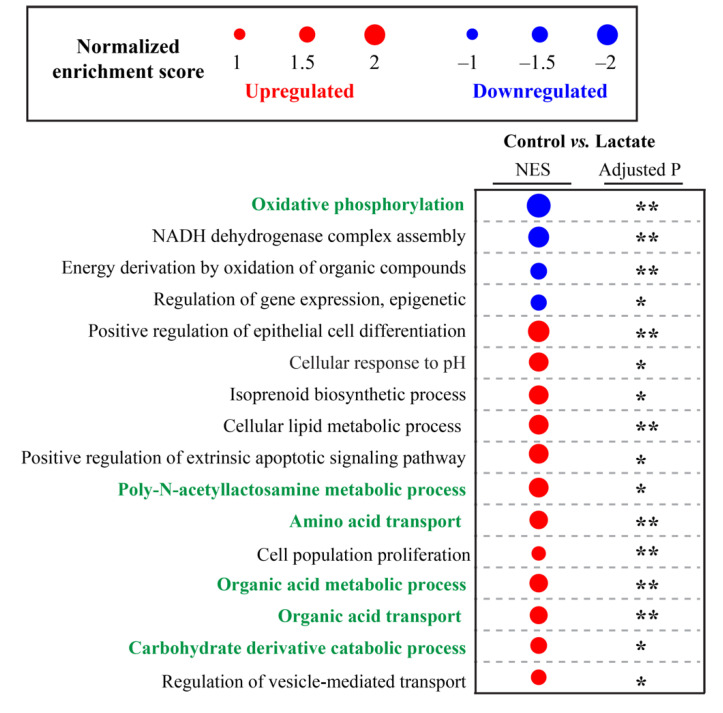
Pathways significantly enriched in lactate treatment compared with control treatment. NES: normalized enrichment score. Negative NES (blue dot) shows downregulated pathways and positive NES (red dot) shows upregulated pathways in lactate treatment, green-highlighted pathways are significantly altered both at the metabolic and transcriptomic levels, * Adjusted *p* < 0.05, ** Adjusted *p* < 0.01.

The heatmap analysis further disclosed altered levels of specific genes between the lactate and control treatment under the major annotated pathways (Figure 5). mRNAs for organic acid transport and metabolism (e.g., *SLC16A3*, *SLC7A8*, and *EPHX1*) were significantly increased by the lactate treatment (Figure 5 and Figure 6, NES = 1.7445, adjusted *p* = 0.002), indicating that colon epithelial cells could quickly respond to and metabolize exogenous lactate.

Notably, mRNAs for oxidative phosphorylation (e.g., *MT-ATP8*, *MT-ND6*, *MT-ND4*, and *MT-CO2*) were significantly decreased by the lactate treatment (Figure 5 and Figure 6, NES = −2.3533, adjusted *p* = 0.0031), which is one of the most prominent roles of mitochondria; to produce the energy currency of the cell, ATP. Along with the downregulation of this process, we observed that lactate treatment significantly upregulated the mRNA expressions involved in phosphorus metabolism (e.g., *ATP6V0C*, *DUSP5*, *ENTPD8*, *RPS6KA4*, and *FKBP8*), organelle organization (e.g., *RTKN*, *FOXA3*, *IFT27*, and *SERPINF2*), and the response to organic substances (e.g., *PTK7*, *SKIL*, *PPP3CA*, and *TOB1*) (Figure 5). These results indicate that lactate could suppress the central function of mitochondria, oxidative phosphorylation, and further re-modulate these downstream processes. Interestingly, lactate treatment significantly activated the mRNA expressions associated with the vesicle-mediated transport (e.g., *CHP1*, *EPN1*, *RAB11B*, and *ADRB1*) (Figure 5 and Figure 6, NES = 1.417, adjusted *p* = 0.016) that occurs from within the cell via the endoplasmic reticulum and Golgi transport (e.g., protein transport) and functions in the endocytosis of material taken into the cell via scavenger receptors. The lactate treatment also significantly upregulated the mRNA expressions for the reproductive process, suggesting the potential role of lactate in epithelial cell proliferation and differentiation. Additionally, lactate treatment significantly enhanced the expression of genes linked to one-carbon metabolism (Figure 5, *MAT1A* and *INMT*), which provides the methyl donor in DNA methylation.

Next, we used the inBio DiscoverTM Database (https://inbio-discover.com/#login, accessed on 26 April 2022) to determine whether any genes altered by lactate treatment could be a potential target for known drugs and gastrointestinal diseases [45]. Figure 5 shows some genes for drug targets (*PPP3CA*, *ADRB1*, and *BRD1*). For example, the *PPP3CA* encoding protein phosphatase 3 catalytic subunit alpha is an important drug target for treating ulcerative colitis with FDA approved drugs (e.g., tacrolimus and cyclosporine), whose expression was upregulated by lactate treatment (Figure 5). Furthermore, some mRNAs associated with gastrointestinal diseases were downregulated by lactate treatment, including *KLF4*, *MT-CO1*, *MT-CYB*, *MT-ND1*, and EPC2, while others were upregulated by lactate treatment, including *PTK7*, *CNOT9*, *FOXA3*, *SERPINF2*, *EPHX1*, *TOB1*, and *PAQR7*. Of these genes, *PTK* (protein tyrosine kinase 2 betas) and *KLF4* (Kruppel-like factor 4) are involved in colon cancer development.

## 3. Discussion

Reactive oxygen species (ROS), a class of reactive molecules and free radicals derived from molecular oxygen, are essential in mediating physiological and pathophysiological signal transduction. Approximately 90% of ROS are generated in the electron transport chain (ETC) of the mitochondria. ROS are generated endogenously as in the process of mitochondrial oxidative phosphorylation [46]. On a physiological level, 0.2–2% of the electrons in the ETC do not follow the standard transfer order but instead directly leak out of the ETC and interact with oxygen to produce superoxide or hydrogen peroxide. The amount determines the beneficial and harmful roles of ROS. A large amount of ROS can cause lipid peroxidation, DNA damage, protein oxidation, irreversible impairment of the mitochondria, insufficient ATP generation, and eventually, cell death. Previous research has indicated that the total cellular ROS levels were elevated after exposure to lactate [47,48,49]. A general perspective regarding the mechanisms of lactate-mediated ROS generation is provided if there exists a high pool of nicotinamide adenine dinucleotide (NADH) during the conversion of lactate to pyruvate that can enter the mitochondria and boost the electron transport chain and high ROS levels [49,50]. 

**Figure 7:**
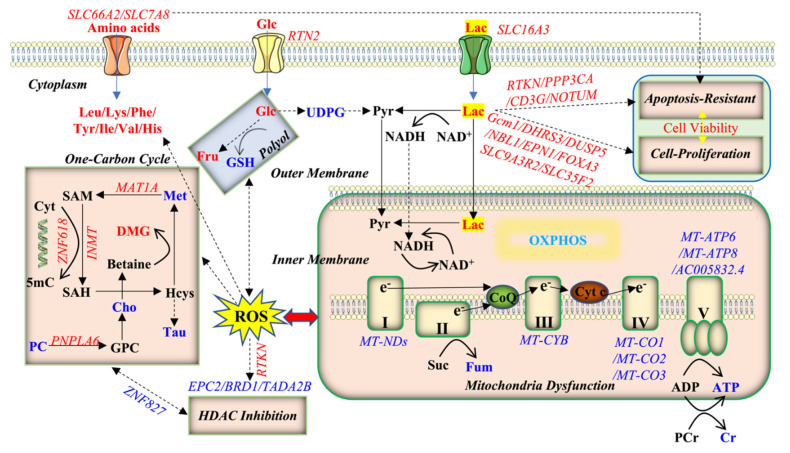
Metabolic and transcriptomic alternations of pathways under lactate exposure. Blue (downregulated) and red (upregulated) in the lactate vs. the control treatments.

Oxidative phosphorylation is the metabolic pathway in which cells produce ATP by enzymes to oxidize nutrients. Cellular ATP generated by oxidative phosphorylation(OXPHOS)through the electron transport chain composed of transmembrane protein complexes (I-V), ubiquinone (Coenzyme Q), and cytochrome c in the cristae of mitochondria [51] is the source of energy for use and storage at the cellular level. Therefore, the reduced ATP production caused by lactate exposure observed in our study was a significant sign of reduced oxidative phosphorylation (Figure 7). In addition, creatine works as the acceptor of phosphate at the end of oxidative phosphorylation in the mitochondria [52]; this was also decreased. Fumarate produced from succinate during the electron transfer process in complex II was found at a low level in the lactate group. The reduction in ATP, creatine, and fumarate suggests a function inhibition of oxidative phosphorylation due to lactate treatment. Consistently, lactate significantly downregulated the levels of mRNAs for complex I (*MT-ND1*, *MT-ND2*, *MT-ND4*, *MT-ND4L*, *MT-ND5*, *MT-ND6*), complex Ⅲ (*MT-CYB*), complex Ⅳ (*MT-CO1*, *MT-CO2*, *MT-CO3*), and complex V (*MT-ATP6*, *MT-ATP8, AC005832.4*) (Figure 7 and Table S1). 

The polyol pathway is known as an accessory glucose pathway that bypasses glycolysis [53]. Hyperglycemia is considered to activate the polyol pathway [54]. In our study, a higher glucose level occurred in the cell cytosol, which is supposed to be induced by the ROS signal pathway (Figure 7). There was no change in the expression of the glucose transporter, *SLC2A4/GLUT4*. However, the expression of *RTN2* was significantly activated by the lactate treatment that plays a role in the translocation of *SLC2A4/GLUT4* from intracellular membranes and facilitates the uptake of glucose into the cell. The expression of *SLC2A4RG*, known as a transcriptional regulator of the *GLUT4* gene, was significantly upregulated by the lactate treatment. In addition, lactate might lead to a high pool of NADH followed by excessive ROS (Figure 7 and Table S1). It has been reported that cellular glucose uptake is stimulated by ROS [55]. Uridine diphosphate glucose (UDPG), a precursor of glycogen, was observed with a decreased level, suggesting that the glycogenesis in which glucose molecules are added to chains of glycogen for storage was also inhibited (Figure 7). In summary, glucose was shunted into the branching polyol pathway to counteract intracellular hyperglycemia.

Sorbitol, fructose, and NADH are the overall products of the polyol pathway [56]. Sorbitol accumulates in primary human monocytes and the human monocytic cell line, MonoMac6, by the stimulation of exogenous lactate [57]. In our study, sorbitol was undetected in our NMR spectra due to its low concentrations. However, we observed a significantly increased level of fructose due to lactate exposure. The change in fructose was approximately 30% of the change in glucose from the polyol pathway in the brain [58]. Similarly, in our study, lactate induced the change in fructose that was equal to 27% of the change in glucose in the Caco-2 cells. In addition, GSH is known to be consumed in the polyol pathway [59]. An increased accumulation of intracellular sorbitol in the polyol pathway [60] may cause toxicity to DNA due to an increase in the intracellular osmotic pressure [61]. Oxidative stress generated through the polyol pathway and aldose reductase competed with glutathione reductase for NADPH. This resulted in decreasing glutathione, as was observed in our study, and increasing intracellular oxidative stress [62].

Taken together, to counteract intracellular hyperglycemia, glucose was shunted into the branching polyol pathway, leading to fructose accumulation. The data indicated lactate-modulated fructose metabolism in human colon epithelial cells via the polyol pathway. However, a high glucose concentration may be toxic to endothelial cells due to apoptosis and the augmentation of oxidative stress [63]. The polyol pathway may contribute to the NADH/NAD^+^ redox imbalance in cells exposed to lactate.

The one-carbon metabolism, comprised of the methionine cycle, folate cycle and choline cycle, is central to cellular function, providing methyl groups for the synthesis of DNA, polyamines, amino acids, creatine, and phospholipids [64]. S-adenosyl-L-methionine (SAM) is the primary methyl donor and is important in regulating gene expression or repression [65]. Methionine adenosyltransferase (MAT) [66], controlled by the *MAT1A* gene, is an essential enzyme that catalyzes the formation of SAM. In our study, a lower level of methionine, the precursor of SAM, and a higher *MAT1A* expression indicated that a rapid generation of SAM was activated by lactate exposure. In addition, alterations in the one-carbon pathway metabolites, including increasing dimethylglycine and decreasing Cho, PC, and Tau, were observed. DNMT, responsible for the transfer of a methyl group from the universal methyl donor, S-adenosyl-L-methionine, to the five-position of cytosine residues in DNA, was not observed with any alternations in our study. However, *INMT*, known as indolethylamine-*N*-methyltransferase, plays a role in transferring one or more methyl groups from the methyl donor SAM to the substrates [67], which was overexpressed by a magnitude of 2.2 due to the lactate treatment. *INMT* is generally involved in the methylation of the tryptamine pathway that is associated with brain activity [68]. In addition, *ZNF618* is a specific reader of 5hmC, an intermediate of DNA demethylation in vivo, and can co-localize with UHRF2. UHRF2 is essential in maintaining DNA methylation in differentiated cells and has been reported to play a crucial role in pooled DNA and histone binding [69]. Therefore, *ZNF618* facilitates UHRF2 chromatin localization [70]. *ZNF618* is among the novel candidate gene products associated with oxidative stress responses [71]. Histone deacetylase (HDAC) activity is a critical modulation pathway, and lactate is linked with endogenous inhibitors of HDAC and promotes changes in gene expression [17]. *ZNF827* was reported to recruit nucleosome remodeling and the histone deacetylation complex. The depletion of *ZNF827* was followed by the loss of cell viability [72]. Previous studies reported the capacity of lactate to inhibit the activity of HDAC only in very high concentrations (IC_50_ = 40 mM) [17,73,74]. However, in our study, a decreased level of *ZNF827* was observed, suggesting that HDAC could be inhibited slightly with a lower concentration of lactate at 8 mM, though this might be not high enough to inhibit HDAC function. *KLF4* has been reported to be upregulated by HDAC inhibitors [75]. However, a significantly decreased expression of *KLF4* was observed without apparent changes in HDAC. The function of downregulated *KLF4* is unknown, but it is supposed to be associated with the HDAC process. The other related transcriptional regulations on *EPC2*, *BRD1*, and *TADA2B* were also observed. 

Mitochondrial ROS production and detoxification are tightly balanced. Shifting this balance enables ROS to activate intracellular signaling and induce cellular damage and cell death. Tauffenberger et al. disclosed that lactate promoted oxidative stress resistance through ROS signaling [47]. Lactate significantly enhances DNA damage repair [76], and incubating cells with lactate will lead to a less compact chromatin structure, facilitating DNA transcriptional machinery, and improving cell survival. Incubating cells with lactate for 24 h evoked a transcriptionally permissive chromatin conformational state and a significant upregulation of essential gene expressions involved in DNA (Figure 7). However, no significant difference was observed in the cell viability in the lactate-induced group, suggesting that some self-protection mechanism against apoptotic distress might exist.

Lactate has been demonstrated to activate TLR4 signaling, NF-κB transcriptional activity, and the expression of inflammatory genes in human U937 histiocytes. *RTKN* is a gene that encodes a scaffold protein that interacts with GTP-bound Rho proteins, mediating Rho signaling to activate NF-κB and it may confer increased resistance to cellular apoptosis in gastric tumorigenesis since NF-κB is known to protect cells against apoptosis [77]. Our study suggests that the upregulation of *RTKN* is responsible for the activation of NF-κB pathway followed by the lactate treatment. We also observed the significant upregulated genes involved in apoptotic protection, including *CD3G*, *NOTUM*, and *PPP3CA* in Caco-2 cells after the lactate treatment (Figure 7 and Table S1), suggesting that lactate is important for cells to be resistant to apoptosis.

A previous study discovered a novel apoptosis-resistant pathway that promoted both immune suppression and oncogenic cell proliferation by modulating the expression of the FOXO family [78], and these are suspected to promote tumor progression. In our study, accompanied with the self-protection against apoptotic activation, a list of mRNAs including *Gcm1* [79], *DHRS3* [40], *DUSP5* [80], *NBL1*, *EPN1*, *SLC9A3R2* [81], *SLC35F2* [82], and *FOXA3* [83] involved in the cell proliferation, were significantly increased by the lactate treatment. Solute carriers (SLCs), ranking second among membrane transport proteins in terms of abundance [84], play crucial roles in human physiology through processes such as the intake of amino acids in the intestine for cell growth [85]. *SLC66A2* and the *SLC7A8/SLC3A2* heterodimer function as an amino acid exchanger [86,87] whose expressions were both upregulated by the lactate treatment with an increased level of amino acids. However, these complicated mechanisms require further investigation.

## 4. Materials and Methods

### 4.1. Caco-2 Cells Culture and the Growth Curve

Caco-2 cells (ATCC #HTB-37) were maintained and cultured in Minimum Essential Medium (MEM) media supplemented with 20% fetal bovine serum, 1% of penicillin-streptomycin, 1× non-essential amino acid solution, and 10 mM of sodium pyruvate at 37 °C and 5% CO_2_.

After subculturing for 24 h, Caco-2 cells were cultured for an additional 24 h prior to treatment with different concentrations (0, 0.78, 3.12, 12.5, 50, 200 mM) of sodium DL-lactate (Sigma-Aldrich). The cell viability was determined using the 3-(4,5-dimethylthiazol-2-yl)-2,5-diphenyl-2H-tetrazolium bromide (MTT) assay as previously described [88].

### 4.2. RNA Sequencing (RNA-Seq)

The Caco-2 cells were treated with 8 mM sodium DL-lactate solution (Sigma-Aldrich) or 8 mM NaCl (control treatment) for 24 h. The total RNA was extracted using the RNeasy Plus Mini Kit (Qiagen). The 1 ng total RNA with 28S/18S >1 and RNA integrity numbers 7.1 to 9.3 were used for the RNA-Seq library construction. cDNA was synthesized using the SMART-Seq HT kit (Takara Bio). The RNA-Seq libraries were constructed with the Nextera DNA Flex Library Prep kit and the Nextera DNA Unique Dual Indexes Set A (Illumina). TapeStation completed the quality control of the libraries (checking the size distribution with an average fragment size of 600 bp when analyzed with a size range of 150–1500) and quantitative PCR (qPCR) assays (a beginning concentration of 2 nM and a final loading concentration of 300 pM). Finally, the libraries were sequenced on NovaSeq6000 (Illumina) at 2 × 150 bp, aiming for 50 million paired end reads per sample.

The RNA sequencing analysis was performed using the CLC genomics workbench (Qiagen 21.0.4). Data were normalized to reads per kilobase of transcript per million mapped reads (RPKM). A false discovery rate (FDR)-corrected *p* value < 0.05 was used as the criterion for significance. A principal component analysis (PCA) was performed to evaluate the outliers of the transcriptomes of all the samples (SIMCA 17.01). A gene set enrichment analysis (GSEA) was performed by iDEP V0.94 using the pre-ranked fgsea method [89]. Heat maps of the differentially expressed genes (DEGs) in the enriched pathways were constructed using supervised hierarchical clustering in Heatmapper [90].

### 4.3. Metabolism Sample Collection and Preparation

The cells were washed with 2 mL of water two times after the removal of medium and quenched with liquid N_2_. Then, cells were stored at −80 °C until they were extracted for the solution-state NMR experiments. The extraction steps were conducted according to the protocol described in the literature [91]. Briefly, 1 mL the extraction solution containing 80% methanol was added to the flask, and the cells were scrapped into a 2 mL EP tube followed by two sonicating and vortex cycles. The mixtures were then centrifuged at 12,000× *g* for 45 min at 4 °C, prior to collecting the supernatant. Methanol was removed using a spin vac, and then water was removed using a freeze-dryer. The dry extracts were stored at −80 °C until NMR detection was conducted.

### 4.4. NMR Experiment and Data Analysis

The extracts were reconstituted in 520 μL of phosphate buffer (0.1 M K_2_HPO_4_: NaH_2_PO_4_, in D_2_O, pD 7.4), and 500 μL of the sample was pipetted into a 5.0 mm NMR tube after the removal of any particulates by centrifugation (5 min, 12,000× *g*, 4 °C). The one-dimensional ^1^H NMR spectra were acquired at 25 °C using an 800 MHz Bruker Avance III NMR system (Bruker BioSpin, Ettlingen, Germany) equipped with a 5 mm TCI cryoprobe. The water-suppressed 1D-NOESY sequence was obtained using a 100 ms mixing time [92]. For each spectrum, 64 scans were collected using a 5 s relaxation delay, 120,190 points, and a 12 kHz sweep width. For assignment purposes, the two-dimensional ^1^H–^1^H J-resolved (J-Res), ^1^H–^1^H correlation (COSY), ^1^H–^1^H total correlation (TOCSY), ^1^H–^13^C heteronuclear single quantum coherence spectroscopy (HSQC), and ^1^H–^13^C heteronuclear multiple bond correlation spectroscopy (HMBC) spectra were acquired for a subset of samples.

The free induction decays were multiplied by a 0.3 Hz exponential window function, Fourier transformed, and manually corrected for phase and minor baseline distortions using Topspin 3.6.1 (Bruker BioSpin, Germany). The right peak of the doublet from lactate with a chemical shift at δ 1.32 was used as a reference. The ^1^H NMR spectra between 0.6 and 9.5 ppm were divided into 2793 spectral peaks with a uniform bucketing (bucket width = 0.002) method using the NMRPROCFLOW online software (https://nmrprocflow.org (accessed on 26 April 2022). The regions containing water (δ 4.65−5.0) were removed to avoid the effects of imperfect water suppression. Additionally, signals from DL-lactate (δ 1.2−1.46 and δ 4.08–4.12) were also excluded from the cell extraction spectra. A probabilistic quotient normalization method [93] was performed for the binning data prior to the multivariate analysis on the dataset scaled to the unit variance by use of MetaboAnalyst [94] (https://www.metaboanalyst.ca/ (accessed on 26 April 2022), a comprehensive platform dedicated to metabolomics data analysis via a user-friendly, web-based interface. The principal component analysis (PCA) was performed on the dataset to generate an overview of the metabolic effects of lactate exposure. Orthogonal projection to the latent structures discriminant analysis (O-PLS-DA) was subsequently performed on the dataset. A cross-validation method [95] and permutation test method [96] were used to certify the validity of the model.

The coefficient plots were generated from the back-transformed loadings incorporated with color-coded coefficients of the loadings plotted in MATLAB (The Mathworks Inc., Natwick, MA, USA, version 7.1) [97]. The loadings coefficients represent the weights of metabolites contributing to the separation between classes. The red color denotes significance in the differentiation between types, while the blue color indicates no value. Based on the number of samples included in each group, correlation coefficients greater than 0.878 were regarded as significant in the discrimination based on a 95% confidence limit.

## 5. Conclusions

In this study, we showed a comprehensive and integrated analysis that lactate modulates the functions in human colon epithelial cells including the inhibition of OXPHOS, the alternation of one-carbon, the activation of the polyol pathway, and cell self-protection against apoptosis, highlighting the multiple roles of lactate in gut–gut microbiota interactions as a signaling molecule. This entire work was based on a cell model in vitro. More thorough investigations are required in an in vivo model in the future.

## Figures and Tables

**Table 1:** ^1^H NMR assignment for the metabolites from the cell extracts.

Number	Metabolites	δ ^1^H (ppm) and Multiplicity	Abbreviation
1	Pantothenate	0.893 (s), 0.927 (s)	PA
2	Isoleucine	0.937 (t), 1.264 (m), 1.46 (m), 1.007 (d), 1.97 (m)	Ile
3	Leucine	0.953 (d), 0.964 (d), 1.705 (m), 3.78 (t)	Leu
4	Valine	0.989 (d), 1.04 (d), 2.259 (m)	Val
5	α-Keto-isovalerate	1.12 (d), 3.01 (m)	α-KIV
6	α-keto-β-methyl-valerate	0.883 (t), 1.088 (d)	α-KMV
7	Lactic acid	1.324 (d), 4.104 (q)	Lac
8	Alanine	1.477 (d), 3.774 (q)	Ala
9	Threonine	1.325 (d), 4.247 (dd)	Thr
10	Glutamate	2.339 (m), 2.052 (m), 2.117 (m), 3.759 (t)	Glu
11	Glutamine	2.453 (m), 2.153 (m), 3.769 (t)	Gln
12	*N*-Acetyl-L-Glutamine	2.037 (s), 2.335 (t)	NAG
13	Succinate	2.399 (s)	Suc
14	Methionine	2.644 (t)	Met
15	Dimethylglycine	2.827 (s)	DMG
16	Creatine	3.032 (s), 3.926 (s)	Cre
17	Choline	3.193 (s)	Cho
18	Phosphocholine	3.218 (s)	PC
19	Glycerophosphocholine	3.227 (s)	GPC
20	Taurine	3.26 (t), 3.41 (t)	Tau
21	Glutathione	2.164 (m), 2.554 (m) 2.931 (dd), 2.975 (dd), 4.55 (m)	GSH
22	Glucose	4.644 (d), 5.23 (d)	Glc
23	Fructose	4.016 (m), 4.032 (m), 4.06 (m)	Fru
24	Galactose-1-phosphate	5.452 (dd)	Gal-1-P
25	Tyrosine	6.899 (d), 7.192 (d)	Tyr
26	Phenylalanine	7.327 (m), 7.425 (m), 7.373 (m)	Phe
27	Fumaric acid	6.515 (s)	Fum
28	Nicotinamide adenine dinucleotide	8.428 (s)	NAD^+^
29	Formate	8.454 (s)	FA
30	Histidine	7.073 (s), 7.826 (s)	His
31	Adenosine monophosphate	8.607 (s), 8.269 (s), 6.147 (d)	AMP
32	Adenosine triphosphate	8.533 (s), 8.269 (s), 6.147 (d)	ATP
33	Uridine 5’-monophosphate	8.116 (d)	UMP
34	Uridine 5’-diphosphate	7.99 (d), 5.968 (d)	UDP
35	UDP Glucuronate	7.952 (d)	UDPG
36	UDP-*N*-acetylglucosamine	7.946 (d)	UDP-*N*-Ace

Note: s, singlet; d, doublet; t, triplet; q, quartet; m, multiplet; dd, doublet of doublet.

## Data Availability

Not applicable.

## Supplementary Materials

The following supporting information can be downloaded at: https://www.mdpi.com/article/10.3390/ijms23116262/s1.

## Abbreviations

Caco-2	Human colon epithelial cells;
NMR	Nuclear Magnetic Resonance;
HDAC	Histone deacetylase activity;
GPR81	G protein-coupled receptor 81;
TNF	Tumor Necrosis Factor;
MTT	3-(4,5-dimethylthiazol-2-yl)-2,5-diphenyl-2H-tetrazolium bromide;
PCA	Principal component analysis;
OPLS-DA	Orthogonal projection to the latent structures discriminant analysis;
SAM	S-adenosyl-methionine;
ROS	Reactive oxygen species;
ETC	Electron transport chain;
NADH	Nicotinamide adenine dinucleotide;
MAT	Methionine adenosyltransferase;
DMNT	DNA methyltransferase;
UHRF2	E3 ubiquitin-protein ligase;
SLCs	Solute carriers;
DEGs	Differentially expressed genes;
NOESY	Nuclear Overhauser effect spectroscopy;
JRES	J-resolved spectroscopy;
^1^H−^1^H COSY	^1^H−^1^H correlation spectroscopy;
^1^H−^1^H TOCSY	^1^H−^1^H total correlation spectroscopy;
^1^H-^13^C HSQC	^1^H-^13^C heteronuclear single quantum coherence spectroscopy;
^1^H-^13^C HMBC	^1^H-^13^C heteronuclear multiple bond correlation spectroscopy;
OXPHOS	Oxidative phosphorylation;
PA	Pantothenate;
Ile	Isoleucine;
Leu	Leucine;
Val	Valine;
α-KIV	α-Keto-isovalerate;
α-KMV	α-keto-beta-methyl-valerate;
Lac	Lactate;
Ala	Alanine;
Thr	Threonine;
Glu	Glutamate;
Gln	Glutamine;
NAG	*N*-Acetyl-Glutamine;
Suc	Succinate;
Met	Methionine;
DMG	Dimethylglycine;
Cre	Creatine;
Cho	Choline;
PC	Phosphocholine;
GPC	Glycerophosphocholine;
Tau	Taurine;
GSH	Glutathione;
Glc	Glucose;
Fru	Fructose;
Gal-1-P	Galactose-1-phosphate;
Tyr	Tyrosine;
Phe	Phenylalanine;
Fum	Fumaric acid;
NAD^+^	Nicotinamide adenine dinucleotide;
FA	Formate;
His	Histidine;
AMP	Adenosine monophosphate;
ATP	Adenosine triphosphate;
UMP	Uridine 5′-monophosphate;
UDP	Uridine-5′-diphosphate;
UDPG	UDP Glucuronate;
UDP-*N*-Ace	UDP-*N*-acetylglucosamine.
